# Multistage Grading of Amnestic Mild Cognitive Impairment: The Associated Brain Gray Matter Volume and Cognitive Behavior Characterization

**DOI:** 10.3389/fnagi.2016.00332

**Published:** 2017-01-10

**Authors:** Caishui Yang, Xuan Sun, Wuhai Tao, Xin Li, Junying Zhang, Jianjun Jia, Kewei Chen, Zhanjun Zhang

**Affiliations:** ^1^State Key Laboratory of Cognitive Neuroscience and Learning & IDG/McGovern Institute for Brain Research, Beijing Normal UniversityBeijing, China; ^2^Beijing Aging Brain Rejuvenation Initiative Centre, Beijing Normal UniversityBeijing, China; ^3^Department of Geriatric Neurology, Chinese PLA General HospitalBeijing, China; ^4^Banner Alzheimer's InstitutePhoenix, AZ, USA

**Keywords:** amnestic mild cognitive impairment, cognition, medial temporal lobe, gray matter volume, voxel-based morphometry

## Abstract

**Background and Purpose:** It is well known that there is a wide range of different pathological stages related to Alzheimer's disease (AD) among patients with amnestic mild cognitive impairment (aMCI). Further refinement of the stages based on neuropsychological and neuroimaging methods is important for earlier disease detection, as well as for the development and evaluation of disease-modifying interventions.

**Materials and Methods:** In this cross-sectional study, 125 aMCI patients were classified into declined progressively three stages of mild, moderate and severe, utilizing the extreme groups approach (EGA) based on their memory function. Fifty-two patients, in addition to 24 cognitively normal subjects, were included in further structural MRI analyses. Characteristics of cognitive functions and brain structures across these newly defined stages were explored through general linear models.

**Results:** Almost all the non-memory cognitive functions showed progressive decline as memory function deteriorated. In addition, medial structures including the right hippocampus, right lingual and left fusiform gyrus, presented with greater gray matter (GM) atrophy during the later stages of aMCI (corrected *p* < 0.05). Correlations were found between GM volume of the lingual gyrus and processing speed (*r* = 0.419, *p* = 0.003) and between the fusiform gyrus and general cognitive function (*r* = 0.281, *p* = 0.046). Moreover, both cognitive function and GM volume presented non-linear trajectories over stages of aMCI.

**Conclusion:** Our study characterized the cognitive profiles along with the degree of episodic memory impairment, and these three stages of aMCI showed non-linear progressive decline in cognitive functions and GM volumes.

## Introduction

Amnestic mild cognitive impairment (aMCI) has been widely viewed as the transitional preclinical stage between normal aging and the onset of Alzheimer's disease (AD). Substantial studies have investigated aMCI from various perspectives, uncovering how strikingly similar its clinical and pathological changes are to those of AD (Mariani et al., 2007; Petersen, 2011; Mufson et al., 2012). Furthermore, as the higher incidence of AD progression (Mitchell and Shiri-Feshki, 2009; Petersen et al., 2014) and faster cortical reduction in AD vulnerable regions (Thompson et al., 2003; Chetelat et al., 2005; Jack et al., 2008; Desikan et al., 2009; Mueller et al., 2010; Okonkwo et al., 2010) present in aMCI cohorts, aMCI has become a focus for the early diagnosis and secondary prevention of AD and indicates that there is a crucial time window for emerging AD-modifying therapies. Increasing evidence points to the fact that the current aMCI inclusion criteria indeed cover a wide spectrum of different pathological stages. While a subset of aMCI patients did not show any detectable structural or functional abnormalities in their brain, others manifested pathological changes comparable with those of AD patients, although similar cognitive impairment was detected. Because of this great variability, the depiction of biomarker trajectories over various disease stages may be imprecise, and thus, disease-modifying intervention would be likely to be underpowered and even impeded by the underlying variability. Therefore, the further refinement of the clinical course of aMCI is of great importance to determine the effective time window for more accurate early diagnosis, prognosis and intervention evaluations.

Studies have begun to focus on the issue of aMCI staging. A dichotomy of early and late MCI was put forward by ADNI and several other groups (Aisen et al., 2010; Ye et al., 2013, 2014; Zhuang et al., 2013; Jessen et al., 2014). With this refinement, AD advancement corresponded better with the severity of brain structural atrophy (Ye et al., 2014). Due to the prominent impairment of episodic memory in aMCI and AD patients (Albert et al., 2011; Gallagher and Koh, 2011; Petersen, 2011) and the attempts of using episodic memory to predict the conversion to AD in aMCI patients or normal elders (Chapman et al., 2011; Didic et al., 2013; Gainotti et al., 2014), the emerging dichotomy was mostly based on whether a person scored 1.0 SD or 1.5 SD below the mean of standard test norms on several episodic memory tests (Ye et al., 2013, 2014; Jessen et al., 2014). Although this dichotomy is sufficient for characterizing monotonic change, it may be impossible to capture some nonlinear trends for cognition or neuroimaging biomarkers.

In this study, we attempted to further refine the staging of aMCI patients based on their episodic memory function and to explore the corresponding characteristics and patterns in their cognitive profiles. We then addressed brain structural alterations over these stages along with declines in cognitive function. Finally, we studied the relationship between cognitive function and brain gray matter (GM) volume during the disease development. Our findings provide further information for understanding the aMCI stages and the disease's progression, and contribute to earlier disease detection and better disease-modifying interventions.

## Methods

### Study cohort

As one of the leading groups, we conducted the Beijing Aging Brain Rejuvenation Initiative (BABRI) investigation on the aging and cognitive impairment of urban elderly people in Beijing, China, and on neuroimaging biomarker identifications for MCI and AD risks. The details of the BABRI project design and the recruitment strategy are described elsewhere (Li et al., 2013). So far, we have collected demographic, neuropsychological and neuroimaging data from 1371 subjects, who (a) were 50–80 years old, (b) had at least 6 years of education, (c) scored 24 or higher on the Chinese version of the Mini-Mental State Examination (MMSE) (Folstein et al., 1975), (d) had no history of neurological, psychiatric, or systemic illness, and (e) had no history of using psychoactive medications.

Among these 1371 subjects, we confirmed a total of 125 subjects who met the published criteria (Petersen, 2011) of amnestic MCI, which included subjective memory complaints, objective memory impairment (scoring more than 1.5 standard deviations below the mean for age- and education-adjusted norms on memory tests), relatively preserved general cognitive function, and intact abilities in their daily life (scoring 0 on the ADL scale).

These 125 aMCI patients were all included in the analyses of cognitive status. For investigating the brain structural changes and their associations with the cognition-based newly introduced aMCI classification, 54 of these aMCI patients were enrolled in the sub-imaging study for the acquisition of structural MRI data, with two being excluded due to image quality. We also included an additional 24 cognitively normal subjects from the BABRI database with available MRI data who were matched for age, gender, and education level.

All study procedures and ethical aspects of this research were approved by the institutional review board of Beijing Normal University Imaging Center for Brain Research. Written informed consent was obtained from each participant.

### Clinical measures

#### Neuropsychological assessment

All our team members who administered the neuropsychological tests and questionnaire were well trained by professional neuropsychologists.

In addition to the MMSE, which was used as a measure of general cognitive function, all the subjects also underwent a battery of neuropsychological tests to assess their cognitive functions, including episodic memory, processing speed, executive function and language ability. Details of these tests can be found in our previous study (Li et al., 2013).

The raw scores of all tests were transformed to a scale ranging from 0 to 10 based on all possible scores (if it had no maximum, the highest observed score plus 1.5 SD of the norms was substituted), as per the following formula:

$$\begin{array}{l} X^{\prime }=([X-X\_min]/[X\_max-X\_min])*10,\\ \text{ if a theoretical maximum existed},\\ X^{\prime }=([X-X\_min]/[(X\_max+1.5\sigma )-X\_min])*10,\\ \text{ if it did not}. \end{array}$$

For the tests scoring the subjects' reaction times, the following formula was utilized:

$$X^{\prime }=10-([X-X\_min]/[(X\_max+1.5\sigma )-X\_min])*10.$$

For a better overall assessment of the performance of certain cognitive functions, the transformed scores within a cognitive domain were averaged to obtain the function scores.

#### Classification of aMCI patients

The memory tests used for the diagnosis of aMCI included long-delay recall (trial 5) and total word recall (trial 1–5) of the R-AVLT and delay recall of the ROCF. Memory impairment was considered if the score was ≥1.5 SD below the mean when compared with the age- and education-adjusted norms.

To classify the aMCI patients into different stages, we used the extreme groups approach (EGA) (Feldt, 1961; Alf and Abrahams, 1975; Preacher et al., 2005) based on memory function scores. Briefly, we computed episodic memory function scores by averaging the transformed memory test scores, then ranked the scores of aMCI patients in descending order and defined the highest 27% as mild aMCI, the lowest 27% as severe aMCI, and those in between as moderate aMCI.

Among the 125 consecutively recruited aMCI patients, there were 33 mild aMCI patients, 57 moderate aMCI patients and 35 severe aMCI patients. The proportion of the three aMCI stages in the sub-imaging study was similar (Chi-square test, *p* = 0.796), with 12 mild, 24 moderate and 16 severe aMCI patients.

### Data acquisition of sub volumetric MRI study

Magnetic resonance imaging data acquisition was performed using a Siemens Trio 3.0 Tesla scanner (Trio; Siemens, Erlangen, Germany) in the Imaging Center for Brain Research at Beijing Normal University. Foam padding and headphones were used to reduce head motion and scanner noise. The patients were instructed to keep still with their eyes closed. The T1-weighted structural images were acquired using three-dimensional (3D) magnetization prepared rapid gradient echo sequences: 176 sagittal slices, a repetition time of 1900 msec, an echo time of 3.44 msec, a slice thickness of 1 mm, a flip angle of 9°, and a field of view of 256 × 256 mm.

### Voxel-based morphometry processing steps

The VBM8-toolbox (http://dbm.neuro.uni-jena.de/vbm) was used to preprocess the structural images with the default parameters, and the MNI East Asian brains template was used for the Affine Regularization. The images were bias-corrected, tissue classified, and normalized to MNI-space using linear (12-parameter affine) and nonlinear transformations, within a unified model (Ashburner and Friston, 2005) including the high-dimensional DARTEL normalization. Gray and white matter segments were modulated only by the nonlinear components to preserve actual GM and WM values locally (modulated GM and WM volumes).

Homogeneity of GM images was checked using the covariance structure of each image with all other images, as implemented in the check data quality function. Two extreme outliers (one in each of the moderate and severe aMCI groups) showing anatomical abnormalities or artifacts were identified and excluded. The remaining *n* = 76 images were free of any QC issues. The modulated GM images were smoothed with a Gaussian kernel of 8 mm full-width-half-maximum (FWHW).

### Data analysis

We first sought to characterize the non-memory cognitive profiles of each stage of aMCI patients by using SPSS 22.0 (SPSS Inc. Chicago, IL, USA). ANOVA and chi-square tests were performed to compare demographic variables among the groups. ANCOVAs with age, gender and years of education as covariates were performed to compare cognitive function scores, and for multiple comparison correction, Bonferroni's *post-hoc* analyses were conducted. Further, general linear models were performed to explore the change trajectories of cognitive function and brain volume, and partial correlation analyses were conducted to identify the relationship between brain volume and cognitive functions, with age, gender and years of education as covariates in above analyses. Thresholds for these analyses were set to *p* < 0.05.

To explore the brain structural differences, smoothed GM images of mild, moderate and severe aMCI, with cognitively normal subjects, were included in the random-effect group analyses, as full-factorial analyses with age, gender and years of education as covariates in SPM8 software (http://www.fil.ion.ucl.ac.uk). Similar analyses within the three aMCI groups were repeated to confirm these different regions. *Post-hoc* pairwise analyses were run to determine which group(s) contributed to the differences. All analyses were performed at the whole brain level. The mean GM volume in those significant regions was then extracted for further analyses. The threshold of significance for all analyses was set at *p* < 0.05 by combining the individual voxel *p* < 0.001 with a cluster size larger than 150 mm^3^, based on Monte Carlo simulations (Ledberg et al., 1998).

## Results

### Demographic data and neuropsychological profiles of the aMCI groups

**Figure 1:**
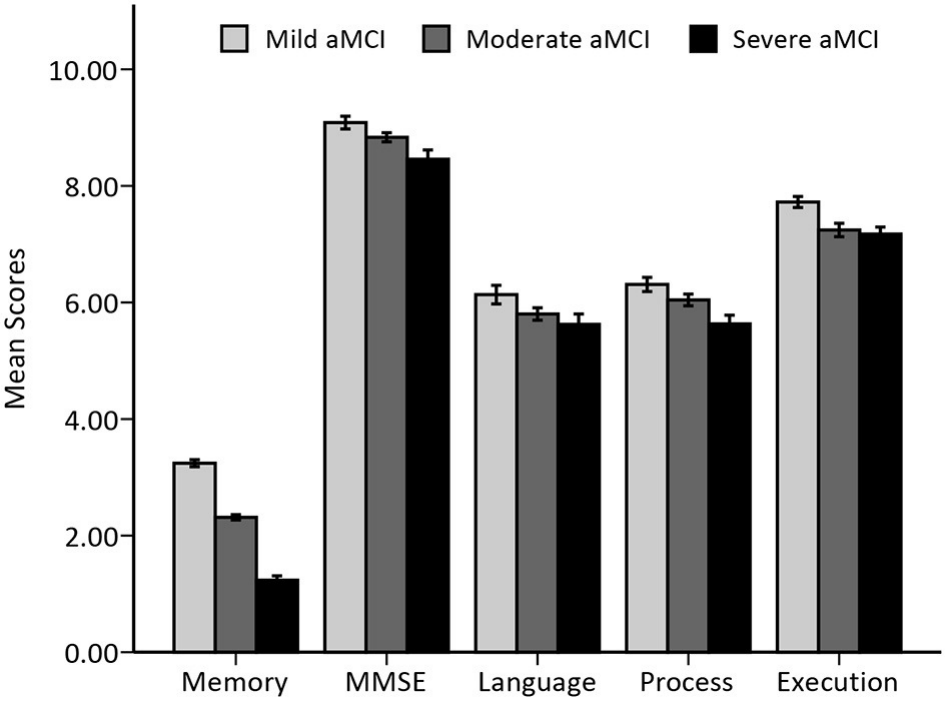
**Histogram of overall cognitive function among the aMCI groups**. Mean and standard errors of cognitive function scores, expressed as transformed and/or composited scores, among three stages of aMCI patients are presented.

There were no differences in the gender ratio or years of education among the three groups. However, the severe aMCI group was significantly older than the mild aMCI group. In the examination of the neuropsychological test scores the moderate and severe aMCI groups showed significantly lower scores on the MMSE and SDMT (both *p* = 0.001) and longer reaction times on part B of the Stroop test (*p* = 0.038) than those in the mild group. Trends for significantly lower scores or longer reaction times were found in other tests. The general cognitive function, processing speed and executive function of the aMCI patients showed progressive declines from the mild group to the severe group, whereas language function only presented a trend of decline (see Table 1 and Figure 1). More specifically, besides the linear decrease in general cognitive function and processing speed (both *p* < 0.001), executive function was found to decline in a sigmoidal pattern (*p* = 0.002), with language function being exponential (*p* = 0.001).

**Table 1:** **Demographic and neuropsychological data of the aMCI population**.

	**Mild aMCI**	**Moderate aMCI**	**Severe aMCI**	***F*-value (χ^2^)**	***P*-value**
**DEMOGRAPHIC**					
No. of Subjects	33	57	35		
Age (years)	60.45 ± 7.10	62.77 ± 5.87	66.51 ± 8.09^†^	6.808	0.002
Years of Education (years)	10.42 ± 2.39	10.79 ± 2.35	11.34 ± 2.98	1.130	0.327
Gender (male/female)	16/17	23/34	15/20	0.566	0.754
**GENERAL COGNITIVE FUNCTION**					
MMSE	27.27 ± 1.77	26.53 ± 1.74	25.20 ± 2.73^†^^‡^	7.645	0.001
MMSE^*^	9.09 ± 0.59	8.84 ± 0.58	8.40 ± 0.91^†^^‡^	7.645	0.001
**EPISODIC MEMORY FUNCTION**					
RAVLT 20 min	2.91 ± 1.10	2.18 ± 1.12^†^	0.46 ± 0.66^†^^‡^	45.947	<0.001
RAVLT (sum of five trials)	19.82 ± 5.35	18.00 ± 4.31	11.77 ± 3.53^†^^‡^	28.939	<0.001
ROCF delayed	14.42 ± 7.01	7.65 ± 4.73^†^	4.97 ± 3.70^†^^‡^	32.475	<0.001
Memory^*^	3.24 ± 0.33	2.31 ± 0.34^†^	1.24 ± 0.41^†^^‡^	233.886	<0.001
**LANGUAGE FUNCTION**					
CVFT	41.18 ± 7.53	37.37 ± 7.10	37.00 ± 7.36	2.816	0.064
BNT	22.45 ± 3.61	21.63 ± 3.75	20.83 ± 4.53	1.744	0.179
Language^*^	6.18 ± 0.88	5.82 ± 0.80	5.66 ± 0.99	2.939	0.057
**ATTENTION/PROCESSING SPEED**					
SDMT	33.32 ± 8.86	29.34 ± 9.32	23.32 ± 7.39^†^^‡^	7.493	0.001
TMT part A, sec	64.12 ± 24.85	65.53 ± 30.90	78.89 ± 28.45	1.234	0.295
Stroop part A, sec	29.79 ± 10.11	31.53 ± 8.15	33.21 ± 10.28	0.547	0.580
Stroop part B, sec	40.61 ± 10.11	45.37 ± 11.63	48.03 ± 14.43^†^	3.357	0.038
Process ^*^	6.31 ± 0.68	6.04 ± 0.75	5.63 ± 0.85^†^	4.135	0.018
**EXECUTIVE FUNCTION**					
TMT part B, sec	179.39 ± 52.03	216.42 ± 82.65	228.06 ± 64.55	2.226	0.112
TMT part B - A, sec	115.27 ± 38.45	150.89 ± 67.32^†^	149.17 ± 57.72	2.989	0.054
Stroop part C, sec	78.30 ± 18.64	91.09 ± 32.78	92.21 ± 29.22	1.767	0.175
Stroop part C - B, sec	37.70 ± 16.68	45.72 ± 28.03	44.18 ± 20.29	0.902	0.409
Execution^*^	7.74 ± 0.53	7.25 ± 0.86^†^	7.20 ± 0.68	3.631	0.030

*Abbreviations: aMCI, amnestic mild cognitive impairment; MMSE, Mini-Mental State Examination; RAVLT, Rey's Auditory Vocabulary List Test; ROCF, Rey-Osterrieth Complex Figure test; CVFT, Category Verbal Fluency Test; BNT, Boston Naming Test; SDMT, Symbol Digit Modalities Test; TMT, Trail Making Test*.

* *Transformed or composited scores are presented. Data are presented as the mean ± SD*.

One-way ANCOVA post-hoc Bonferroni tests are indicated by abbreviations:

† p < 0.05, when compared to mild aMCI;

‡ *p < 0.05, when compared to moderate aMCI. Age, gender and years of education are covariates*.

To ensure that the aMCI subpopulation in the neuroimaging sample was representative of the aMCI cohort, we compared their demographic and neuropsychological profiles by independent *t*-tests. As expected, there were no statistically significant differences (data not shown). When we compared the aMCI subpopulation with the CNs, aMCI patients showed significant declines in all cognitive domains (see Supplementary Table 1, Supplementary Figure 1).

### VBM data–group comparison for the imaging sample

**Figure 2:**
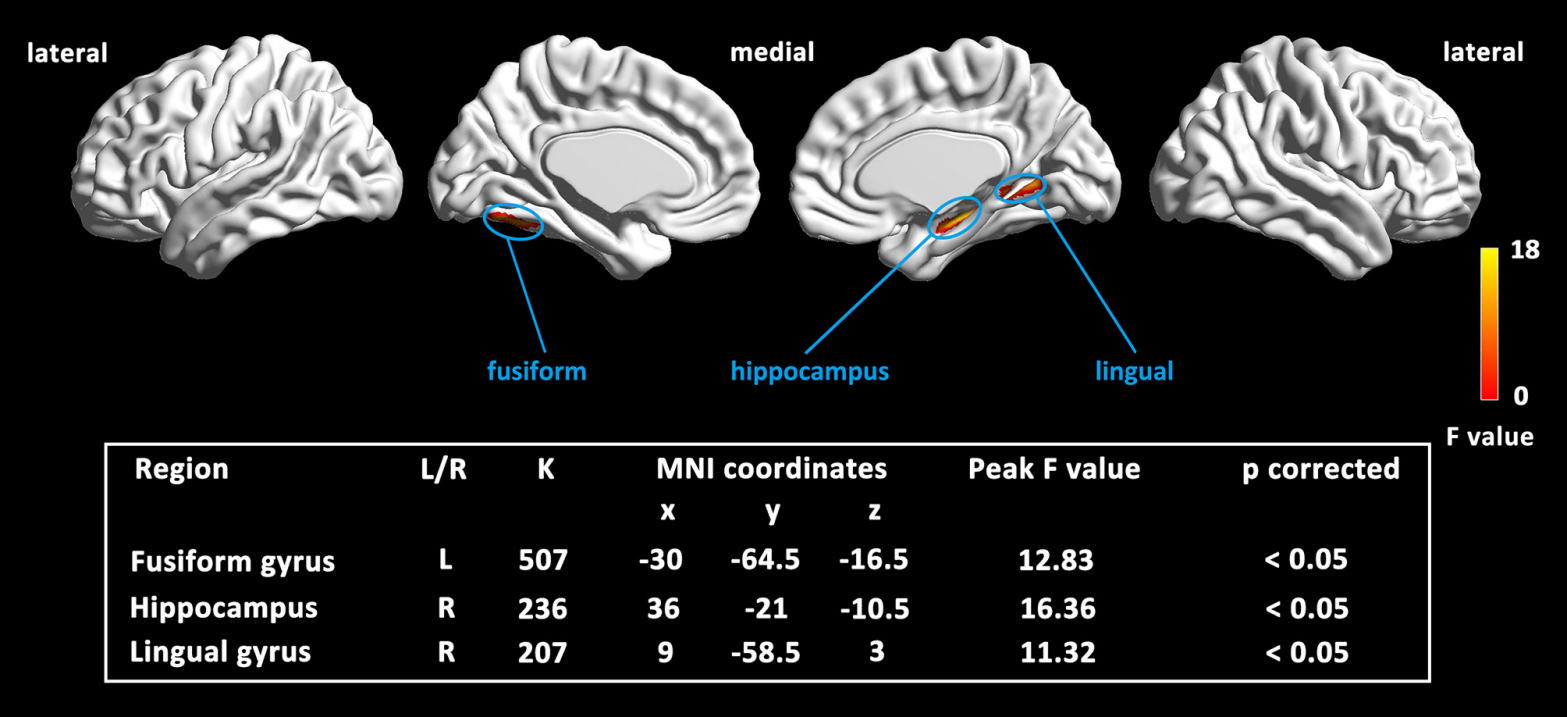
**Map of the significantly different regions in GM volume among three aMCI stages**. Results were based on threshold at *p* < 0.05 by combining the individual voxel *p* < 0.001 with a cluster size larger than 150 mm^3^, using Monte Carlo simulations (Ledberg et al., 1998).

**Figure 3:**
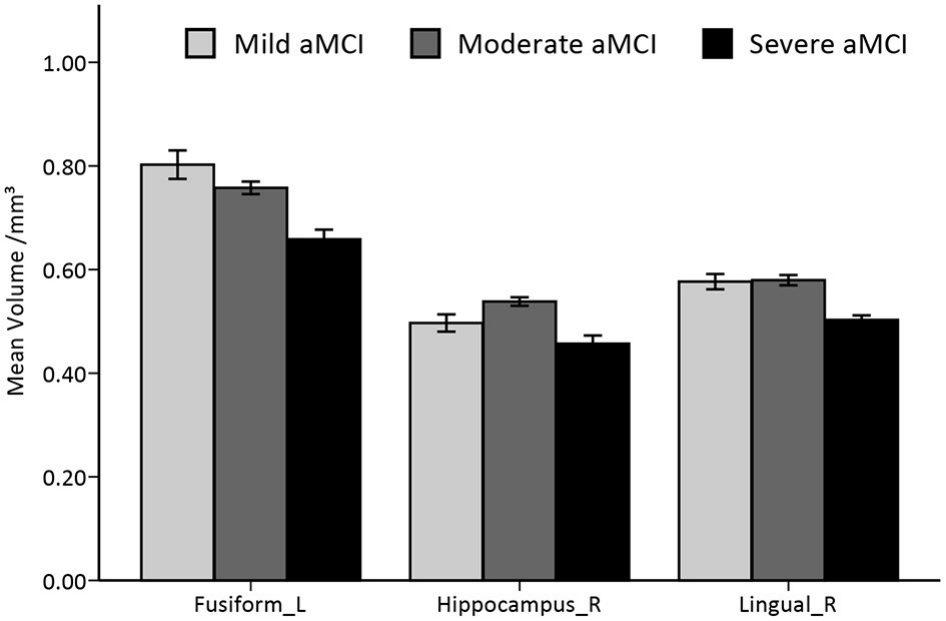
**Histogram of volumetric differences of significant regions in VBM analyses among the three stages of aMCI**. Mean and standard errors of volumes in significant ROIs are presented. Significant differences are only found in comparing the severe stage to the mild/moderate stage of aMCI.

The full-factorial analyses revealed several areas where the local GM volume showed significant differences among the newly introduced stages of aMCI, including the typical vulnerable regions of AD in the medial temporal lobe. Similar results were found in the analyses among aMCI groups, combined or not with the cognitively normal group (see Figure 2 and Supplementary Figure 2). The right hippocampus and the lingual gyrus, extending to parahippocampal gyrus and limbic structures, showed prominent differences, with notable differences in the left fusiform gyrus as well. Further pairwise analyses revealed that the considerable structural atrophy of the severe aMCI group contributed to all significances in comparison with the regional volume (see Figure 3).

Among the aMCI groups, the mean GM volume alteration in these ROIs were then fitted by general linear models. An exponential model was found to best fit the volume changes in the left fusiform gyrus (*R*^2^ = 0.36, standard β = −0.60, *p* < 0.001). When the analyses were conducted on the other two ROIs from the quadratic models that fit the data better (*R*^2^ = 0.39 for the lingual gyrus, *R*^2^ = 0.32 for the hippocampus, both quadratic coefficients <0, both *p* < 0.001), we found that the peaks of these two curves fell around the mild stage (see Supplementary Figure 3).

### Correlations between non-memory cognitive function and the GM volume

**Figure 4:**
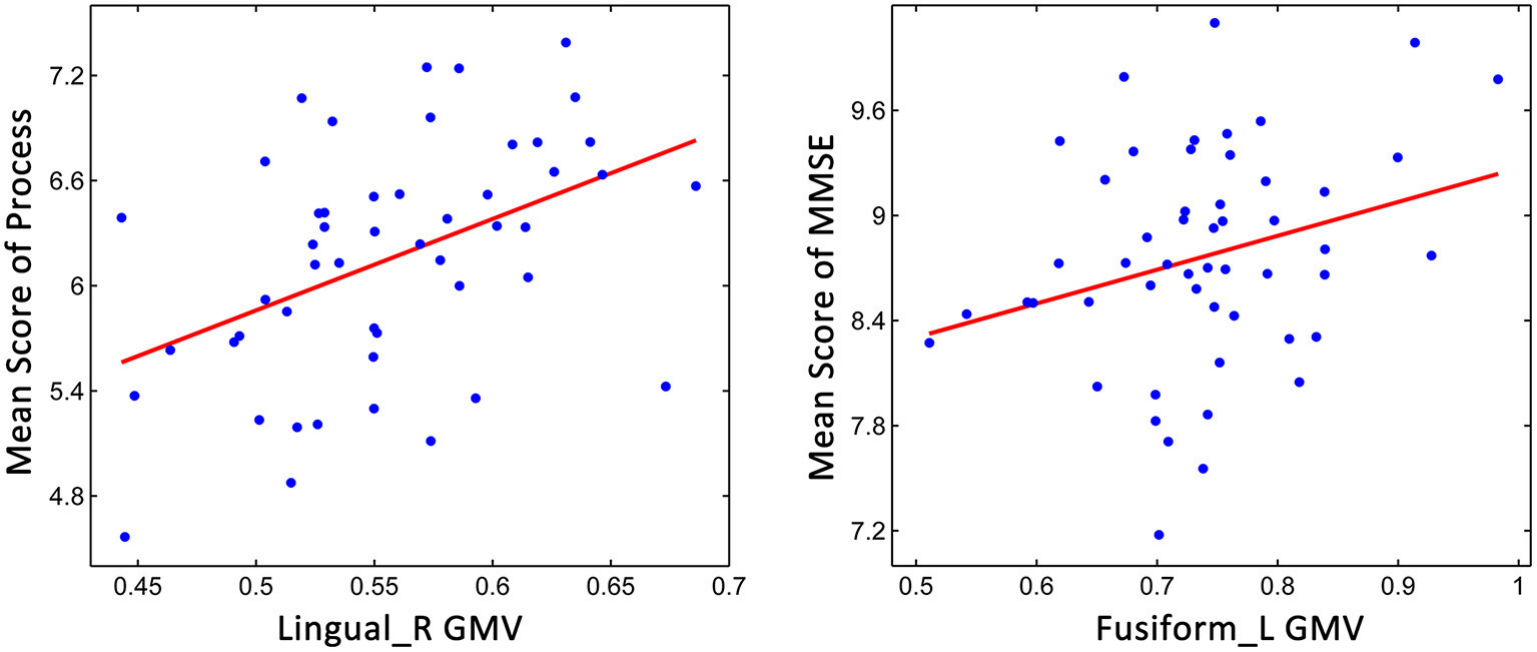
**Relationship between the GM volume measures and scores on non-memory cognitive domains**.

Because the aMCI classification was based on memory function, partial correlation analyses were conducted for the non-memory cognitive functions. As shown in Figure 4, we found that processing speed was significantly correlated with the GM volume of the right lingual gyrus (*r* = 0.419, *p* = 0.003), and significant correlations were also found between general cognitive function and the GM volume of the left fusiform gyrus (*r* = 0.281, *p* = 0.046), indicating that the decline of cognitive functions might relate to the brain structural changes during aMCI progression.

## Discussion

In the present study, we introduced a new way to categorize aMCI patients as mild, moderate or severe based on their degree of episodic memory dysfunction. Within a cohort of aMCI patients, we found comprehensive functional decline along with deterioration of memory function. Brain degenerative changes, as expected, were more evident and extensive during the later stages of aMCI. Furthermore, with this multi-staging aMCI definition, certain non-linear trajectories of these cognitive and GM changes could be characterized and were indeed uncovered by our current study. Notably, we also found significant correlations between cognitive function and brain structural alterations.

Evidence from recent studies reflected a greater need for early anti-AD interventions for earlier stage aMCI patients. This study is our attempt to categorize aMCI patients into three stages based on their episodic memory function in the hope that we are more likely to observe the actual and progressive changes en route to AD and that it would become feasible to depict the non-linear trajectory if it does exist, rather than an artificial linear one.

There are other ways of dividing aMCI patients into only two stages, as presented in other studies. The Alzheimer's Disease Neuroimaging Initiative (ADNI) has recruited a group of subjects with less severe memory impairment than its original MCI cohort and referred them as in the earlier stage of MCI (E-MCI) (Aisen et al., 2010), whereas other studies have defined the E-MCI patients as those with memory scores between −1.5 and −1.0 SD below the age- and education-matched norms (Jessen et al., 2014; Ye et al., 2014) or those who have not converted to AD in a follow-up after 2 years (Zhuang et al., 2013). Although the longitudinal method seems to be the best way to classify the aMCI cohort, it may not be practical.

Episodic memory dysfunction has been considered as the earliest and most common representation in aMCI due to AD, and it has been more deteriorative during the progression toward AD (Albert et al., 2011; Petersen, 2011; Braskie and Thompson, 2013). Recent studies have paid attention to the longitudinal cognitive changes in aMCI progression. Episodic memory was reported to manifest significantly higher rate of decline in aMCI patients who developed AD (Albert et al., 2007; Jack et al., 2008), and it was among the cognitive functions that showed the earliest and fastest decline during a follow-up of aMCI (Wilson et al., 2011). The significant, consistent and characteristic memory decrease in the disease progression is indeed a typical perspective that can be more easily observed and retested.

Together with episodic memory dysfunction, the non-memory cognitive function of aMCI patients showed a similar progressive decline in our study, indicating that the progression of aMCI was probably accompanied by systematic cognitive degeneration. We further found that executive function and language function declines were both relatively slower in the later phase. These findings are consistent with several previous longitudinal studies of aMCI (Albert et al., 2007; Wilson et al., 2011) patients, which indicated that executive function and working memory decline were accelerated earlier in aMCI converters during follow-ups.

Changes in cortical GM volume were also identified based on the three-stage definition in the present study. Regional reduction was presented in the fusiform gyrus during the mild disease stage, and more regions, such as the bilateral hippocampus and right lingual gyrus extending to limbic area, were affected later. Moreover, most of these brain regions exhibited nonlinear but monotonic reductions in GM volume.

Though the current study is cross-sectional, our results generally agreed with previous longitudinal studies concerning the spatiotemporal pattern of cortical reduction in aMCI progression. Cortical atrophy was first shown in the medial temporal lobe, including the anterior hippocampus and fusiform gyrus, and then in the rest of temporal lobe, the temporo-parietal association cortices and the frontal lobe during the conversion of aMCI (Whitwell et al., 2007; Spulber et al., 2012; Yao et al., 2012). These results fit well with the Braak stage, with the earliest neuropathological changes occurring in the anterior medial temporal lobe and fusiform gyrus and then spreading to the neocortex (Brettschneider et al., 2015). In addition to the widespread cortical reduction, spatiotemporal acceleration and/or deceleration of atrophy in the medial temporal structures was also found during the aMCI progression, including the hippocampus, parahippocampal gyrus, entorhinal cortex, lingual gyrus, fusiform gyrus, posterior cingulate gyrus, precuneus/cuneus and so on (Thompson et al., 2003; Chetelat et al., 2005; McDonald et al., 2009; Sabuncu et al., 2011; Tosun et al., 2011). The consistency between these longitudinal findings and our cross-sectional results shows that our newly introduced three-stage definition for aMCI is informative and meaningful.

The relationship between GM volume reduction and cognitive decline was examined in this study. Except for episodic memory, processing speed and general cognitive function were correlated with brain structures. These findings are consistent with studies concerning the cognitive changes in aMCI cohorts (Mcdonald et al., 2012; Yao et al., 2012), as executive function and processing speed are likely linked to posterior cortical regions.

There were some limitations in the present study. First, although size of our aMCI cohort was relatively large to examine the patterns of cognitive changes, the association of cognition and brain structure needs further confirmation due in part to the smaller sample size in the MRI sub-study. Second, despite the inclusion criteria and staging methods for our aMCI groups, it was likely that some of our patients did not present due to AD pathology. Finally, we noted that our nonlinear characterization was mostly restrained to a quadratic model for depicting the trajectories of GM volume alterations. Longitudinal studies would have greater advantages in achieving a better fit with cognitive and cortical data, thus accurately determining whether patterns of regional longitudinal atrophy are different between aMCI patients who have advanced or not.

## Conclusions

Our study characterized the cognitive profiles and brain structures along with the degree of episodic memory impairment, as defined by the three-part staging introduced for aMCI patients. These three aMCI stages showed progressive decline in cognitive functions and reduced GM volumes in regions such as the hippocampus, fusiform and lingual gyrus, which had been previously reported in aMCI patients. More aMCI neuropsychological and neuroimaging data are needed in future longitudinal studies to identify the patterns of cortical atrophy along with cognitive decline.

## Ethics statement

Before the study, all the application materials should be sent to the institutional review board at the Imaging Center for Brain Research at Beijing Normal University and wait for the authorization. And the study cannot be started until the materials have been approved by the ethic committee. If there's any need to change the study protocol or informed consent, all the changes should be reported to the institutional review board at the Imaging Center for Brain Research at Beijing Normal University. To every subject or his/her statutory agent, the researchers are bound to introduce the object, process and potential profits and risks. All subjects should be informed the right to decide whether to participate and whether to quit from this study. Researchers should have the signed informed consent before the study and archive all the informed consent as the research data.

## Author contributions

Guarantors of integrity of entire study, ZZ; Study concepts/design, ZZ; Data acquisition, all authors; Literature research, CY, XS, and WT; MCI diagnosis, XS, XL, and JZ; Statistical analysis, CY, WT, and XL; Drafting of the manuscript, CY, XS; Critical revision of the manuscript for important intellectual content, KC, JJ, and ZZ.

## Funding

This study is supported by the State Key Program of National Natural Science of China (Grant No. 81430100), the Beijing New Medical Discipline Based Group (Grant No. 100270569) and the National Natural Science Foundation of China (Grant No. 30873458 and 81173460).

### Conflict of interest statement

The authors declare that the research was conducted in the absence of any commercial or financial relationships that could be construed as a potential conflict of interest.
