# Supplementary material for: Multistage Grading of Amnestic Mild Cognitive Impairment: The Associated Brain Gray Matter Volume and Cognitive Behavior Characterization
Source: Front Aging Neurosci. 2017 Jan 10;8:332. doi: 10.3389/fnagi.2016.00332 (PMC5222841; doi:10.3389/fnagi.2016.00332)
Supplement: Supplementary file 1 [file Table1.DOCX]

Supplementary Material

**Multistage Grading of amnestic mild cognitive impairment: the associated brain gray matter volume and cognitive behavior characterization**

**Caishui Yang^1,2^, Xuan Sun^3^, Wuhai Tao^1,2^, Xin Li^1,2^, Junying Zhang^1,2^, Jianjun Jia^3^, Kewei Chen^4,2^ and Zhanjun Zhang^1,2*^**

*** Correspondence:**

Dr. Zhanjun Zhang

E-mail: [zhang_rzs@bnu.edu.cn](mailto:zhang_rzs@bnu.edu.cn).

**1. Supplementary Figures and Tables**

**1.1. Supplementary Figures**

**Supplementary Figure 1.**

Histogram of overall cognitive function among the neuroimaging sample. Raw mean and standard errors of cognitive function scores, expressed as transformed and/or composited scores, amongst the cognitively normal and aMCI groups are presented.

**Supplementary Figure 2.**

Map of the significantly different regions in GM volume among the neuroimaging sample. Results were based on threshold at p < 0.05 by combining the individual voxel p < 0.001 with a cluster size larger than 150 mm^3^, using Monte Carlo simulations (Ledberg et al., 1998).

**Supplementary Figure 3.**

Non-linear trend of GM volume alteration among three aMCI stages. Left: exponential changes in left fusiform gyrus (R^2^ = 0.36, standard β = -0.60, p < 0.001); Right: quadratic changes in right lingual gyrus (R^2^ = 0.39, quadratic coefficients < 0, p < 0.001).

**1.2. Supplementary Tables**

**Supplementary Table 1.**

Demographic and neuropsychological data of neuroimaging sample

|  | CN | Mild aMCI | Moderate aMCI | Severe aMCI |
| --- | --- | --- | --- | --- |
| No. of subjects | 24 | 12 | 24 | 16 |
| Age (years) | 61.21±4.05 | 61.00±6.95 | 63.58±6.11 | 65.88±8.57 |
| Education (years) | 10.67±2.24 | 10.33±2.77 | 11.42±2.45 | 12.25±3.17 |
| Gender (male/female) | 8/16 | 6/6 | 8/16 | 7/9 |
| Number of APOE ε4 carrier * | 4 | 2 | 6 | 3 |
| Memory † | 5.48±1.15 | 3.12±0.23 ‡ | 2.36±0.33 ‡ § | 1.28±0.36 ‡ § |
| MMSE † | 9.69±0.39 | 9.14±0.41 ‡ | 8.78±0.55 ‡ | 8.46±0.74 ‡ § |
| Language † | 7.10±0.75 | 6.31±0.64 ‡ | 5.79±0.83 ‡ | 5.97±0.86 ‡ |
| Process † | 6.96±0.39 | 6.36±0.73 ‡ | 6.26±0.62 ‡ | 5.81±0.77 ‡ |
| Execution † | 8.35±0.25 | 7.66±0.69 † | 7.24±0.91 ‡ | 7.35±0.56 ‡ |

Abbreviations: CN, cognitively normal; aMCI, amnestic mild cognitive impairment.

* Part of APOE genotype data is unavailable.

† Transformed or composited scores are presented. Data are presented as the mean ± SD.

One-way ANCOVA post-hoc Bonferroni tests are indicated by abbreviations: ‡ p<0.05, when compared to CN; § p<0.05, when compared to mild aMCI. Age, gender and years of education are covariates.

**Reference**

Ledberg, A., Akerman, S., and Roland, P.E. (1998). Estimation of the probabilities of 3D clusters in functional brain images. *Neuroimage* 8**,** 113-128.
