# Supplementary figures and images for: Multistage Grading of Amnestic Mild Cognitive Impairment: The Associated Brain Gray Matter Volume and Cognitive Behavior Characterization
Source: Front Aging Neurosci. 2017 Jan 10;8:332. doi: 10.3389/fnagi.2016.00332 (PMC5222841; doi:10.3389/fnagi.2016.00332)

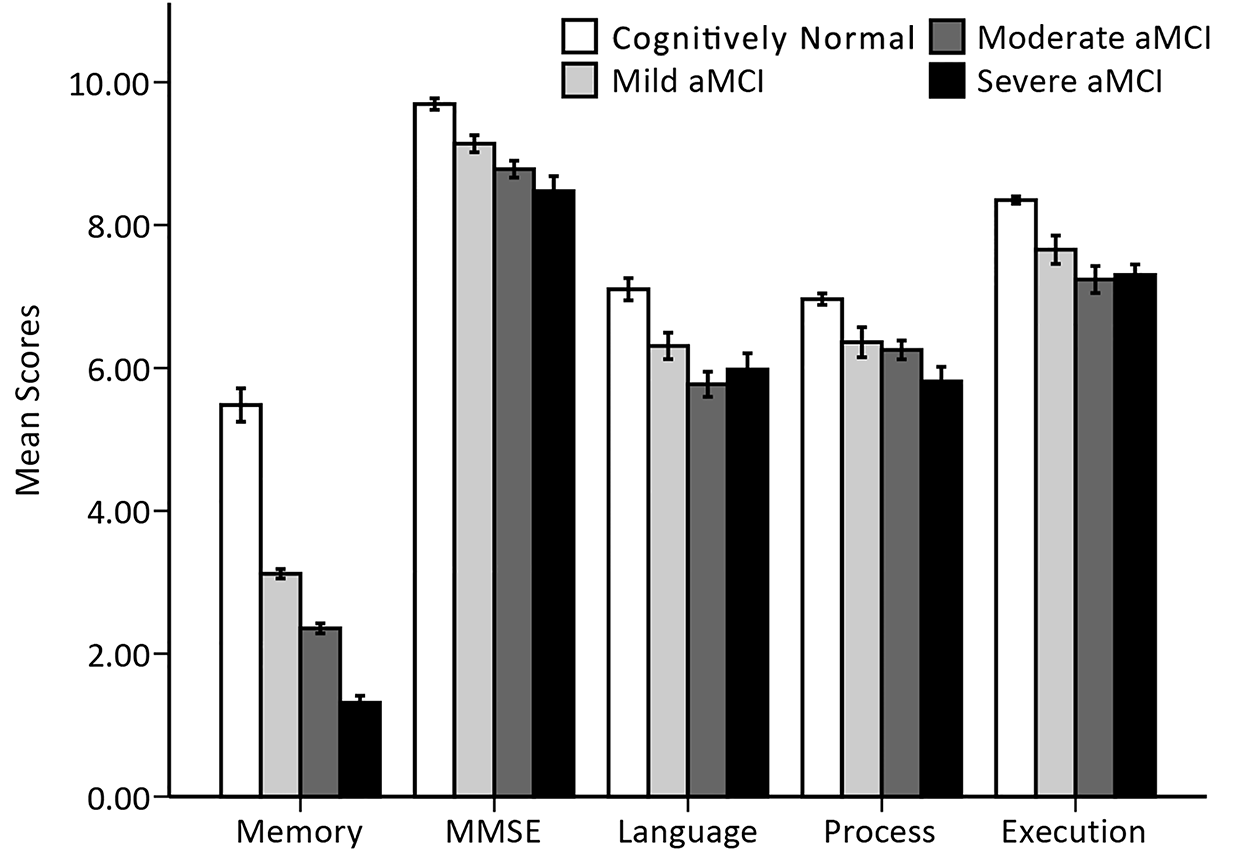

Supplement: Supplementary file 2 [file Image1.TIF]

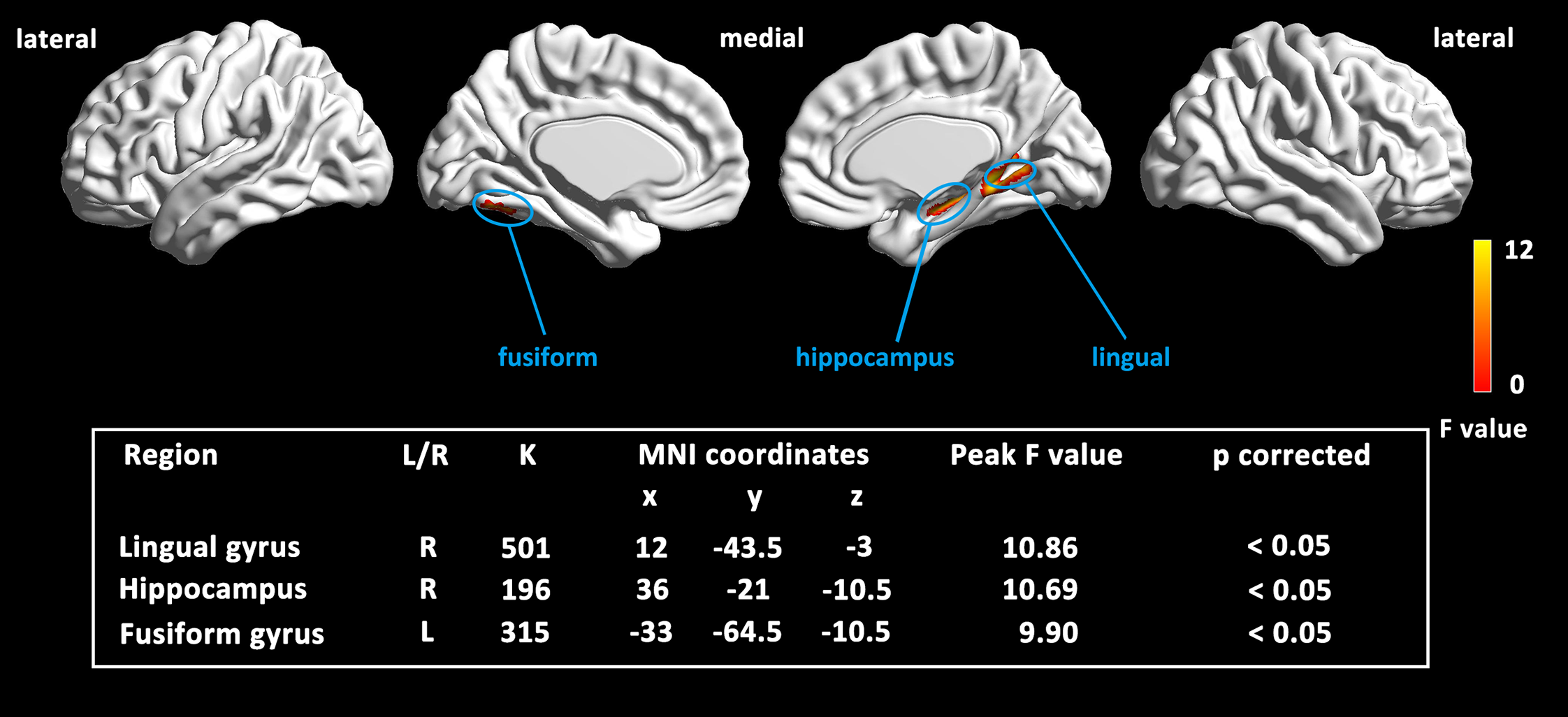

Supplement: Supplementary file 3 [file Image2.TIF]

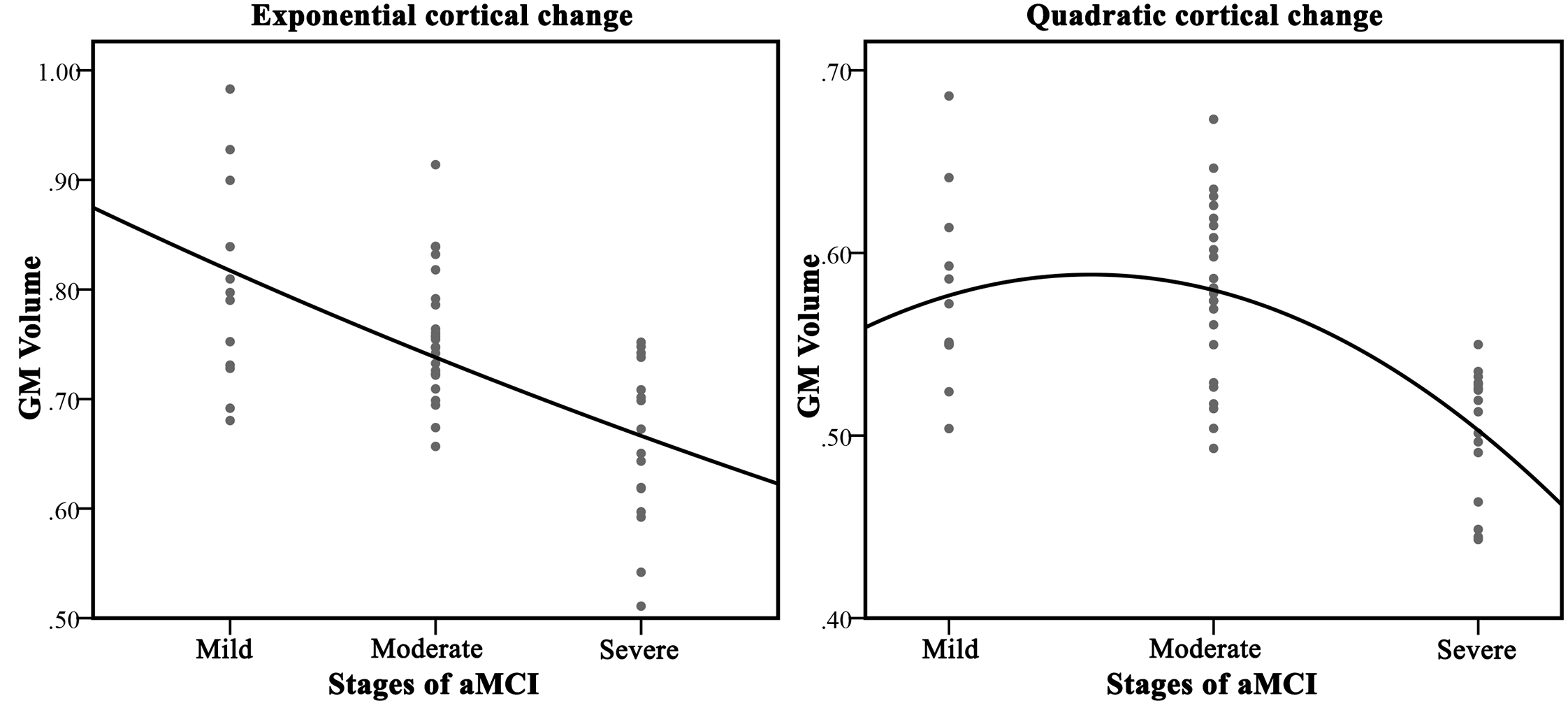

Supplement: Supplementary file 4 [file Image3.TIF]
